# Characteristic of Molecular Subtypes in Lung Squamous Cell Carcinoma Based on Autophagy-Related Genes and Tumor Microenvironment Infiltration

**DOI:** 10.1155/2022/3528142

**Published:** 2022-09-13

**Authors:** Jinjie Wang, Jiaqi Zhu, Yijie Tang, Anping Zhang, Tingting Zhou, Youlang Zhou, Jiahai Shi

**Affiliations:** ^1^Nantong Key Laboratory of Translational Medicine in Cardiothoracic Diseases, and Research Institution of Translational Medicine in Cardiothoracic Diseases, Affiliated Hospital of Nantong University, Medical School of Nantong University, Nantong 226001, Jiangsu, China; ^2^Department of Thoracic Surgery, Affiliated Hospital of Nantong University, Medical School of Nantong University, Nantong 226001, Jiangsu, China; ^3^Research Center of Clinical Medicine, Affiliated Hospital of Nantong University, Medical School of Nantong University, Nantong 226001, Jiangsu, China; ^4^School of Public Health, Nantong University, Nantong 226019, Jiangsu, China

## Abstract

**Background:**

Recently, a large number of studies have sought personalized treatment for lung squamous cell carcinoma (LUSC) by dividing patients into different molecular subtypes. Autophagy plays an important role in maintaining the tumor microenvironment and immune-related biological processes. However, the molecular subtypes mediated by autophagy in LUSC are not clear.

**Methods:**

Based on 490 LUSC samples, we systematically analyzed the molecular subtype modification patterns mediated by autophagy-related genes. The ssGSEA and CIBERSORT algorithm were utilized to quantify the relative abundance of TME cell infiltration. Principal component analysis was used to construct autophagy prognostic score (APS) model.

**Results:**

We identified three autophagy subtypes in LUSC, and their clinical outcomes and TME cell infiltration had significant heterogeneity. Cluster A was rich in immune cell infiltration. The enrichment of EMT stromal pathways and immune checkpoint molecules were significantly enhanced, which may lead to its immunosuppression. Cluster B was characterized by relative immunosuppression and relative stromal activation. Cluster C was activated in biological processes related to repair. Patients with high APS were significantly positively correlated with TME stromal activity and poor survival. Meanwhile, high APS showed an advantage in response to anti-PD1 and anti-CTLA4 immunotherapy.

**Conclusion:**

This study explored the autophagy molecular subtypes in LUSC. We also discovered the heterogeneity of TME cell infiltration driven by autophagy-related genes. The established APS model is of great significance for evaluating the prognosis of LUSC patients, the infiltration of TME cells, and the effect of immunotherapy.

## 1. Introduction

Lung cancer ranks second among common cancers in the world, after breast cancer. The mortality rate of lung cancer is still impressive, accounting for about one-tenth of diagnosed cancers and one-fifth of deaths in 2020 worldwide [1, 2]. Lung squamous cell carcinoma (LUSC), as a major subtype of non-small cell lung cancer (NSCLC), is generally considered to be closely related to smoking [3]. Although, with the help of imaging development and physical examination, great progress has been made in the early diagnosis and treatment of lung cancer, the survival rate of lung cancer is still very low [4, 5]. In addition, due to the heterogeneity of individuals and the insensitivity to molecular targeted therapy, the treatment of LUSC still has great limitations [6]. Therefore, it is urgent to seek new treatment directions and individualized treatment plans for LUSC.

Autophagy is a strictly regulated multistep lysosomal degradation pathway in eukaryotes. It degrades damaged organelles, unfolded proteins, and harmful toxins and transports them to the lysosome for digestion to promote the metabolism and renewal of the cell itself [7]. The imbalance of this regulation process often leads to the disorder of the microenvironment, which may lead to the occurrence of tumor. Autophagy plays the role of double-edged sword in the field of tumors [8]. On the one hand, in the initial stage of cancer, autophagy achieves quality control by removing damaged organelles and toxic components of certain proteins, thereby limiting the transmission of carcinogenic signal pathways and playing a role in suppressing cancer [9]. On the other hand, once the tumor progresses to an advanced stage, cancer cells maintain their metabolism and proliferate migration through autophagy-mediated circulation, thereby promoting the development of the tumor [10]. This indicates that regulating autophagy may become one of the effective intervention strategies for cancer treatment.

Tumor microenvironment (TME) refers to the complex and constantly changing ecosystem in which tumor cells are located. It includes stromal cells, fibroblasts, and endothelial cells around the tumor, as well as natural and adaptive immune cells [11, 12].

The components of TME play a key role in tumor growth and immune response, and their heterogeneity may mediate different immune activation states [13]. Therefore, assessing the status of TME may help us predict the potential response of immunotherapy, so as to carry out separate treatment options.

In this study, we successfully divided the LUSC patients into three autophagy clusters based on autophagy-related genes (ARGs). We then explored the different autophagy modification patterns and the characteristics of TME cell infiltration in the autophagy clusters. In addition, LUSC patients were separated into three gene clusters based on differentially expressed genes (DEGs) identified from the three clusters. At the same time, an autophagy prognostic score (APS) model was established to quantify the autophagy characteristics of individuals and predict the clinical response of patients to immune checkpoint inhibitor (ICI) treatment. These findings indicated that APS can be used as an indicator for prognostic evaluation and selection of immunotherapy for LUSC patients.

## 2. Materials

### 2.1. Data Collection and Processing of the LUSC Cohort

We searched available public databases TCGA (https://portal.gdc.cancer.gov/) and GEO (https://www.ncbi.nlm.nih.gov/geo/) for gene expression, mutation information, and related clinical data of LUSC. Patients with incomplete survival information were excluded. For TCGA data, we obtained RNA-sequencing data (FPKM value) of 490 LUSC patients. The FPKM value was then transformed into Transcripts Per Kilobase Million (TPM) value, which is more comparable to microarray datasets from GEO. Moreover, we downloaded the LUSC microarray datasets with complete clinical information including GSE73403 (*n* = 69), GSE74777 (*n* = 107), and GSE157011 (*n* = 484) from GEO for verification. R (version 4.0.2) was used for data analysis.

### 2.2. Establishment of Autophagy Clusters in LUSC Patients

Initially, 232 ARGs were downloaded from the Human Autophagy Database (HADb: https://www.autophagy.lu/) [14]. The expression of ARGs in LUSC patients was extracted from TCGA. Univariate Cox analysis was then used to screen out ARGs associated with prognosis of LUSC (*P* < 0.05). Then, based on the expression of these genes, unsupervised cluster analysis was performed on LUSC patients to identify different autophagy clusters. The potential number of clusters was determined by the cumulative distribution function (CDF) curve of the consensus score [15]. When using the ConsensusClusterPlus R package to perform the above steps, 1000 repetitions were undertaken to ensure classification stability [16].

### 2.3. Functional Annotation in Autophagy Clusters

Gene set variation analysis (GSVA) was performed by GSVA R package to analyze the biological differences among different autophagy clusters [17]. The “c2.cp.kegg.v6.2.-symbols” gene sets were download from MSigDB database to run the GSVA analysis. A *P* value less than 0.05 was considered statistically significant.

### 2.4. Estimation of TME Cell Infiltration in LUSC Patients

The single-sample gene set enrichment analysis (ssGSEA) algorithm was utilized to quantify the relative abundance of TME cell infiltration in each LUSC patient, which was then normalized to unity distribution from 0 to 1 [18]. To further quantify the specific composition ratio of immune cells in LUSC patients, the CIBERSORT R package was used for specific quantification [19]. The percentages of 22 distinct immune subsets in each LUSC patient and each cluster were then obtained. Then, through the ESTIMATE R package, we calculated the immune score, stromal score, and estimate score of each LUSC patient to predict the level of immune cell and stromal cell infiltration in the tumor, which form the basis for inferring tumor purity [20].

### 2.5. Establishment of Autophagy Gene Clusters

The limma R package was utilized to determine DEGs between autophagy clusters. The identification standard was adjusted: *P* value <0.01 and |logFC| > 1. Then the DEGs associated with the prognosis of LUSC were further determined through univariate Cox analysis (*P* < 0.01). Then, according to the expression of these prognostic-related DEGs, unsupervised cluster analysis was performed again on LUSC patients to identify autophagy gene clusters.

### 2.6. Construction and Evaluation of the APS Model

To quantify the autophagy characteristics of every individual patient, we constructed a comprehensive scoring system called APS. The expression of these prognostic-related DEGs was subjected to the principal component analysis (PCA), in which principal component 1 and principal component 2 were both extracted to construct APS. The advantage of this method is to keep the main components as much as possible while reducing the dimensionality and eliminate the mutual influence factors between the components. After obtaining the expression of each gene, we defined the APS using a method similar to the study of Zhang et al. [21]:

(1) $$\begin{array}{c} \text{APS}=\sum \left(\text{PC}1i+\text{PC}2i\right), \end{array}$$

where *i* is the expression of prognostic-related DEGs.

According to the best cut-off value determined by survminer R package, LUSC patients were divided into high APS group and low APS group. At the same time, the univariate and multivariate Cox regression models including age, gender, stage, and APS were utilized to assess the prognostic factors of LUSC patients. The hazard ratio (HR) value distinguished the prognostic predictors of risk factors and protective factors (HR > 1 was a risk factor and HR < 1 was a protective factor, *P* < 0.05). To verify whether the APS model has wide applicability, three GEO datasets GSE73403, GSE74777, and GSE157011 were downloaded as validation sets. Each LUSC patient had an APS value calculated by this model, and the Kaplan–Meier curve was utilized to reflect its survival value.

### 2.7. Correlation of APS Characteristics with Tumor Mutation Burden (TMB) and Immunotherapy

In order to determine the relationship between APS and TMB, the total numbers of mutations in high APS group and low APS group were calculated, respectively. The maftools R package was used to draw the mutation waterfall plot of the top 20 genes [22]. Meanwhile, the overall mutation rates of the two groups were calculated to obtain the TMB score. After that, the correlation between APS and TMB was analyzed, and their survival curves were drawn, respectively. In addition, to further study the significance of APS in immunotherapy, Wilcoxon test was utilized to explore the differential expression in immune checkpoints such as PD-L1 and CTLA4 among different APS groups. Finally, data of immunophenoscore (IPS) of the immune checkpoint inhibitor (ICI) were download from The Cancer Immunity Database (TCIA, https://tcia.at/home), which can predict the intergroup differences in response to immunotherapy [23, 24].

### 2.8. Statistics

In this study, Wilcoxon test was utilized to analyze the differences between two groups. Kruskal-Wallis and one-way ANOVA tests were utilized to analyze the differences between three or more groups. Spearman analysis was utilized for correlation analysis. The surv_cutpoint function module in the survival R package was utilized for the best grouping. Besides, the survminer R package was utilized to test all potential cut-off points to get the maximum rank statistic. Log-rank test and Kaplan–Meier test were utilized to draw the survival curve.

## 3. Results

### 3.1. Landscape of Prognostic-Related ARGs in LUSC

**Figure 1:**
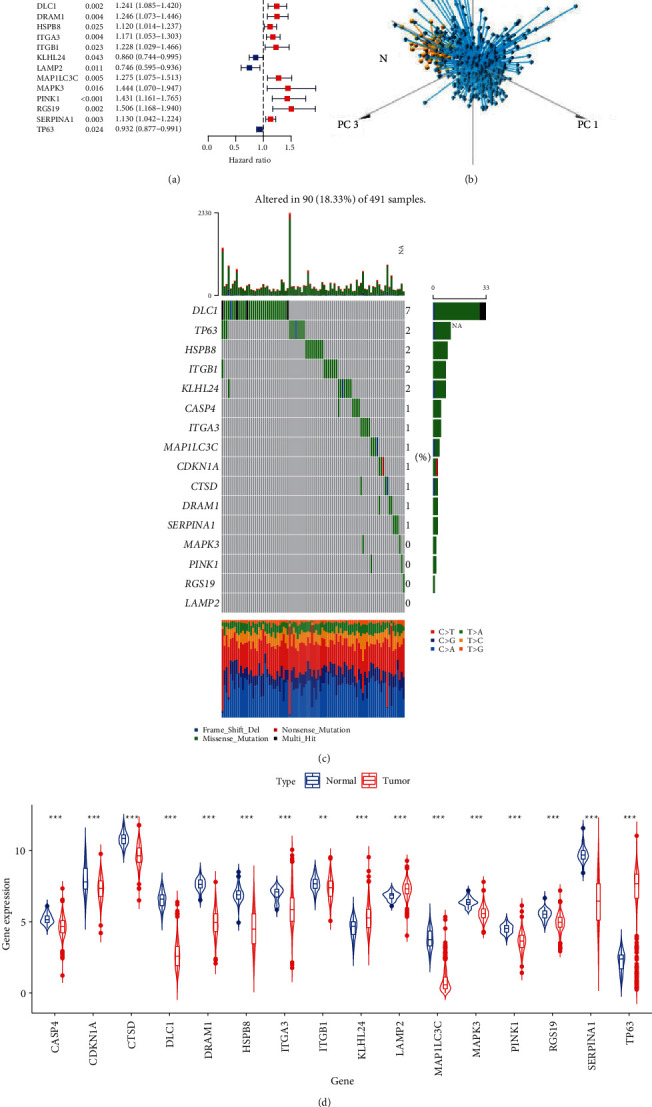
Landscape of autophagy-related genes (ARGs) associated with prognosis of LUSC. (a) Forest plot showing the prognostic values of 16 prognostic-related ARGs in LUSC (*P* < 0.05). (b) Principal component analysis (PCA) for the prognostic-related ARGs between tumor and normal showed separate populations. (c) The waterfall plot showing the mutation landscape of prognostic-related ARGs. (d) The relative expression of prognostic-related ARGs in LUSC.

A total of 210 ARGs were extracted from the TCGA database, among which 16 ARGs were associated with the prognosis of LUSC (Figure 1(a)). By reducing the dimensions of these prognostic-related ARGs, tumor and normal samples showed separate populations (Figure 1(b)). Then, the total mutation of these prognostic-related ARGs was observed in 90 out of 491 LUAD samples (18.33%). It was also pointed out that DLC1 had the highest mutation frequency, followed by TP63 and HSPB8 (Figure 1(c)). At the same time, there were significant differences in the expression of these genes in tumor samples and normal samples, with the exception of TP63, KLHL24, and LAMP2, which were all downregulated in tumors (Figure 1(d)). The above analysis indicated that the expression of these prognostic-related ARGs in normal and LUSC tissues was highly heterogeneous, which may be one of the mechanisms that mediate tumorigenesis.

### 3.2. Identification of Autophagy Clusters in LUSC

**Figure 2:**
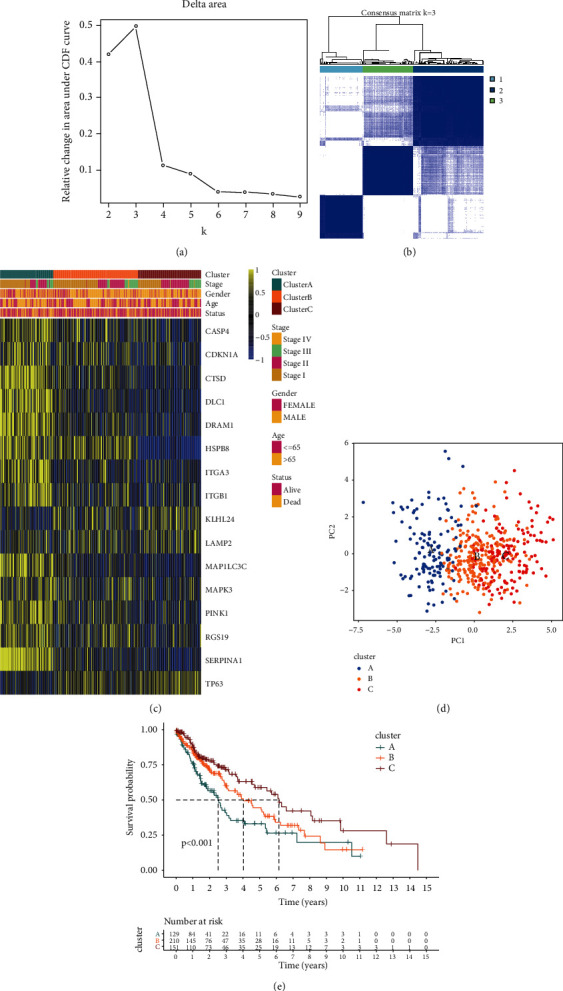
Identification of autophagy clusters in LUSC. (a) Delta area curve for clustering, representing the relative change in area under the cumulative distribution function (CDF) curve. (b) Heat map of sample clustering under *k* = 3 in LUSC. (c) Unsupervised clustering of 16 prognostic-related ARGs in LUSC. Blue, orange, and red represent autophagy clusters A, B, and C, respectively. Cluster, stage, gender, age, and survival status were used as patient annotations. Yellow and blue represent high and low expressions of ARGs, respectively. (d) PCA for the ARGs of three clusters among LUSC patients. (e) Survival analysis of the three autophagy clusters among all LUSC patients in TCGA.

Depending on the expression of above prognostic-related ARGs, unsupervised cluster analysis was performed on LUSC patients. Based on the CDF curve, we selected three clusters as our classification (Figures 2(a) and 2(b), Figures S1A–S1D), and all LUSC patients were then divided into three different clusters (Figure 2(c)). There were 129 cases in cluster A, 210 cases in cluster B, and 151 cases in cluster C. Besides, we found that the ARGs expression in cluster A significantly exceeded those in the other two clusters (Figure S1E). Principal component analysis (PCA) further confirmed the distinct characteristics of these three clusters (Figure 2(d)). At the same time, survival analysis showed that cluster A had a significant survival disadvantage compared with the other two clusters, while cluster C had a prominent survival advantage (Figure 2(e)).

### 3.3. TME Cell Infiltration and Immune Cell Characteristics among Three Autophagy Clusters

**Figure 3:**
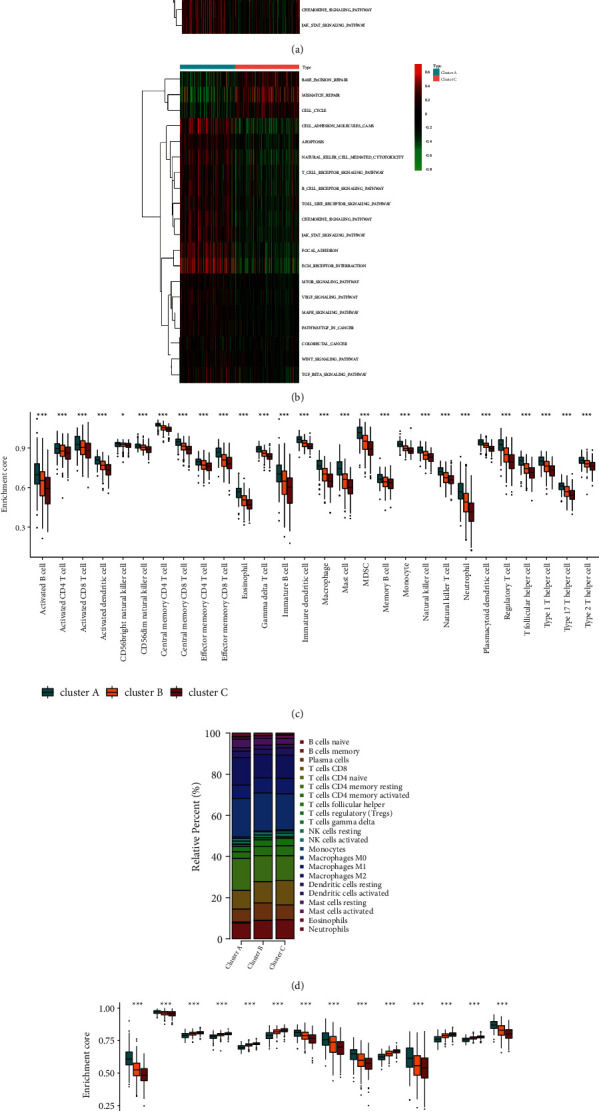
TME cell infiltration and immune cell characteristics in the autophagy clusters. ((A) and (B)) GSVA analysis showing the biological pathways in the three autophagy clusters. Yellow represents activated pathways and blue represents inhibited pathways. (a) Cluster A versus cluster B; (b) cluster A versus cluster C. (c) TME cell infiltration of the three autophagy clusters. (d) The relative percentage of 22 immune infiltrating cells among the three clusters. (e) Differences in stroma-activated pathways among the three clusters. The asterisks represent the *P* value (^*∗*^*P* < 0.05; ^*∗∗*^*P* < 0.01; ^*∗∗∗*^*P* < 0.001).

To further explore the biological differences between the three autophagy clusters, we performed GSVA enrichment analysis in pairs (Figures 3(a) and 3(b)). Cluster A was strongly enriched in pathways associated with immune activation such as T cell receptor, B cell receptor signaling pathway, and natural killer cell mediated cytotoxicity. Clusters B and C were both enriched in base excision repair, mismatch repair, and cell cycle. Moreover, cluster C showed stronger immunosuppression. Next, ssGSEA was used to detect the infiltration of TME cell in the autophagy clusters. The results showed that the overall TME cell infiltration of cluster A was significantly abundant and the overall TME cell infiltration of cluster C was the lowest. Cluster B was somewhere in between (Figure 3(c)). However, patients in cluster A did not show the survival advantage brought by abundant TME cells. The composition of different immune cell may constitute different immune microenvironment, thereby mediating different immune responses. So we further analyzed the specific components of immune cells in the three clusters. The relative percentages of 22 immune cells in each LUSC patient were obtained through CIBERSORT R package (Figure S2A). The composition of immune infiltrating cells among the three autophagy clusters also showed heterogeneity (Figure 3(d)). Cluster A had higher percentages of CD4 memory resting T cells and M2 macrophages compared to the other two clusters. Meanwhile, cluster C was rich in M1 macrophages and activated mast cells. Cluster B had more M0 macrophages and activated dendritic cells compared to the other two clusters. Then we performed a comprehensive score based on the content of the immune cells and stromal cells and found that cluster A was at a relatively high level (Figures S2B–S2D). In addition, we were surprised to find that cluster A not only had a high level of immune cell infiltration but also had strong TME mesenchymal activities such as Epithelial-Mesenchymal Transition (EMT), pan-fibroblast transforming growth factor *β* response (Pan-F-TBRs), and angiogenesis pathways. It was worth noting that cluster A was significantly higher than the other two clusters in terms of immune checkpoint. Meanwhile, cluster C showed higher activity in biological pathways related to repair such as base excision repair, DNA damage repair, and mismatch repair (Figure 3(e)).

### 3.4. Reclassification of Autophagy Subtypes

**Figure 4:**
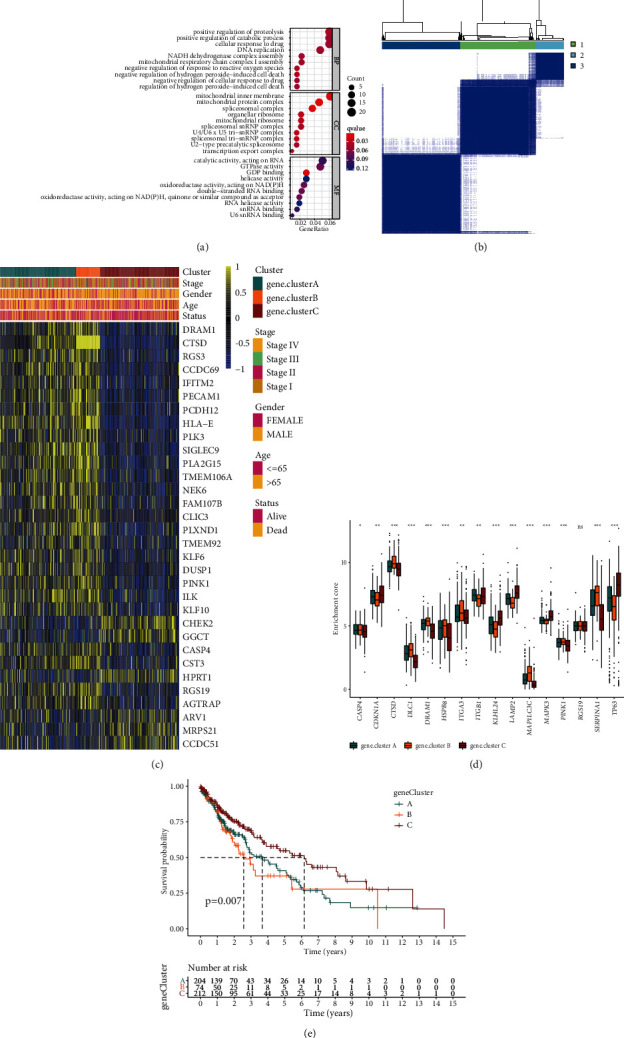
Reclassification of autophagy subtypes in LUSC. (a) Gene ontology analysis of the differentially expressed genes (DEGs). The size of bubbles represents the count of gene enrichment, and the color represents the q value. (b) Heat map of sample clustering under *k* = 3 in LUSC. (c) Unsupervised clustering of prognostic-related DEGs in LUSC. Blue, orange, and red represent gene clusters A, B, and C, respectively. Gene cluster, stage, gender, age, and survival status were used as patient annotations. Yellow and blue represent high and low expressions of prognostic-related DEGs, respectively. (d) The expression of prognostic-related ARGs among the three gene clusters. (e) Survival analysis of the three gene clusters among all patients.

To further explore autophagy modification pattern in LUSC, 373 DEGs were then identified among the three autophagy clusters (Figure S3A). Gene ontology enrichment analysis showed that these DEGs were involved in the positive regulation of proteolysis, mitochondrial inner membrane, and catalytic activity, acting on RNA (Figure 4(a)). Then, the DEGs associated with the prognosis of LUSC were screened out, and unsupervised cluster analysis was performed again on 490 LUSC patients. The reclassification of autophagy subtypes was defined as autophagy gene clusters, of which 204 cases belong to gene cluster A, 74 cases belong to gene cluster B, and 212 cases belong to gene cluster C (Figures 4(b) and 4(c), Figures S3B–S3F). Similarly, these three gene clusters can clearly separate LUSC (Figure S3G). It was worth noting that these gene clusters also have heterogeneity in the expression of prognostic-related ARGs (Figure 4(d)). Among them, gene cluster C showed a low level of expression in most prognostic-related ARGs. The survival curve also suggested that gene cluster C had a clear survival advantage (Figure 4(e)).

### 3.5. Construction and Evaluation of the APS Model

**Figure 5:**
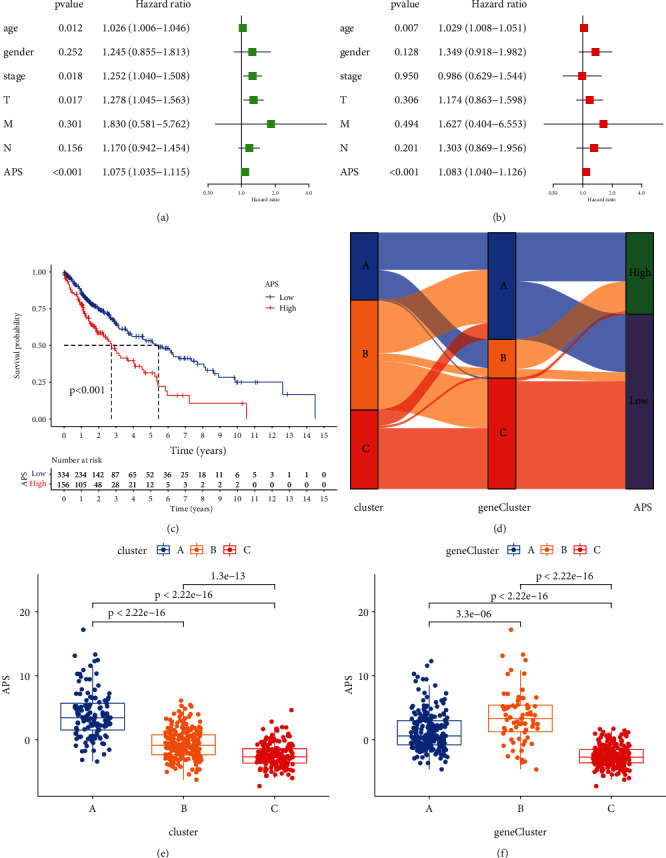
Construction and evaluation of APS model. (a) Univariate Cox regression analysis for the age, gender, TNM status, and APS signature in LUSC patients. (b) Multivariate Cox regression analysis for the age, gender, TNM status, and APS signature in LUSC patients. (c) Kaplan–Meier curves were used to analyze the overall survival of LUSC patients with high (156 cases) and low (334 cases) APS. (d) Autophagy cluster, gene cluster, and APS were associated through alluvial diagram. (e) Difference of APS among autophagy clusters in LUSC. (f) Difference of APS among gene clusters in LUSC.

Considering that the autophagy subtype is only an assessment of different groups of LUSC, it is impossible to accurately assess the autophagy characteristics of every single patient. Therefore, we constructed the APS model to quantify the autophagy characteristics of each LUSC patient. We classified age, gender, TNM status, and APS of every LUSC patient into univariate and multivariate Cox regression analysis (Figures 5(a) and 5(b)). We can conclude that APS can be served as an independent prognostic factor of LUSC. Next, we tried to further verify the value of APS in predicting the prognosis of LUSC patients. According to the cut-off value of 0.935 determined by the survminer R package, LUSC patients were divided into low APS group (*n* = 334) and high APS group (*n* = 156). The survival benefit of patients in the low APS group was significant (Figure 5(c)). The GSE73403 and GSE157011 cohorts further verified that the APS model had a good predictive ability (Figures S4A and S4B). However, in the GSE74777 cohort, although the low APS group had a better prognostic advantage, it was not statistically significant (*P* > 0.05, Figure S4C). Next, we associated autophagy clusters, gene clusters, and APS through alluvial diagram (Figure 5(d)). Most patients in cluster C belonged to gene cluster C and both had low APS. Meanwhile, most patients in gene cluster B belonged to cluster A and had high APS. After that, APS was calculated on different autophagy clusters and gene clusters, which showed a great difference (Figures 5(e) and 5(f)). The scores of cluster C and gene cluster C were both at the lowest level, while the scores of cluster A and gene cluster B were at a high level. The analysis of matrix-related pathway activity indicated that low APS may be closely related to repair activation, while high APS was enriched in EMT and immune checkpoint (Figure S4D). In addition, LUSC patients with low APS had higher survival rates (Figure S4E), while surviving patients had lower APS (Figure S4F). Combining APS with the patient's TNM staging, we can see that, with the increase of T and N, the APS value showed a decreasing trend (*P* < 0.05, Figures S4B–S4C). Stage and M also had the same trend, but it was not statistically significant (*P* > 0.05, Figures S4A and S4D).

### 3.6. Correlation between APS and TMB

**Figure 6:**
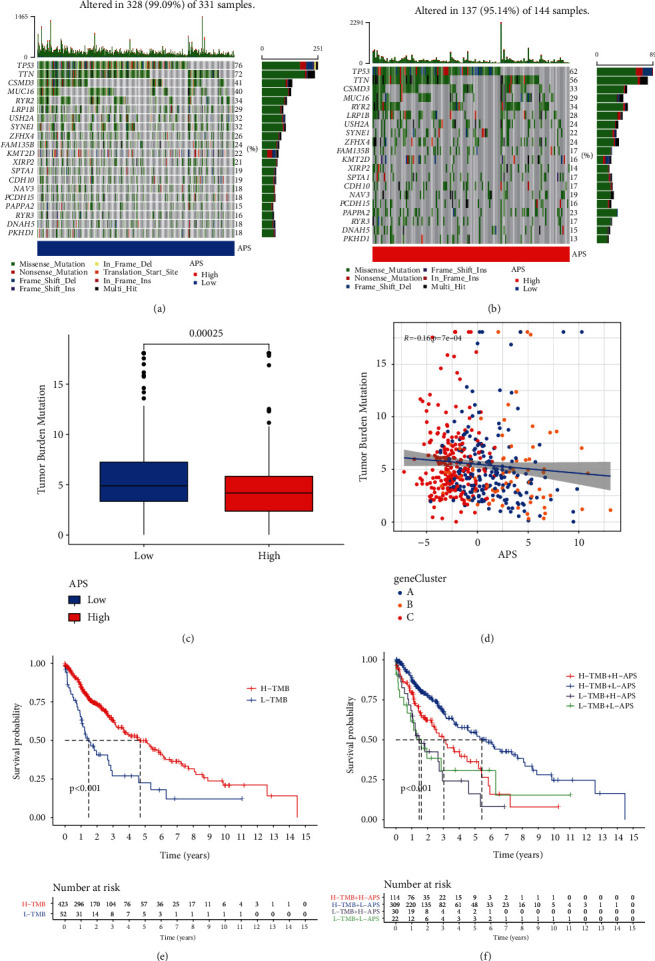
Correlation between APS and tumor mutation burden (TMB). (a) The waterfall plot shows the frequency of tumor somatic mutations landscape in low APS group. (b) The waterfall plot shows the frequency of tumor somatic mutations landscape in high APS group. (c) The relative score of TMB in low and high APS groups. (d) Correlation between APS and TMB. (e) Kaplan–Meier curves of overall survival rates in low and high TMB score groups. (f) Kaplan–Meier curves of overall survival rates by both APS and TMB.

Previous studies have shown that TMB has guiding significance in helping patients choose immune therapy [25, 26]. In view of the differential enrichment of immune checkpoint pathway in different APS groups, we tried to associate APS with TMB. We separately analyzed the tumor somatic mutations of LUSC patients in both high and low APS groups and found that TP53 and TIN had high mutation rates in both groups. In general, the overall mutation rate in the low APS group was higher than that in the high APS group (Figures 6(a) and 6(b)), and the TMB was also at a higher level (Figure 6(c)). Correlation analysis showed a negative correlation between APS and TBM (Spearman coefficient: *R* = −0.16, *P*=7*e* − 04; Figure 6(d)). Next, we jointly studied the impact of TBM and APS on the survival of LUSC. As shown in Figure 6(e), the overall survival rate of patients with high TMB was higher than that of patients with low TMB. At the same time, patients with low APS combined with high TMB had the best survival advantage (Figure 6(f)).

### 3.7. The Effect of APS in Predicting Immunotherapy

**Figure 7:**
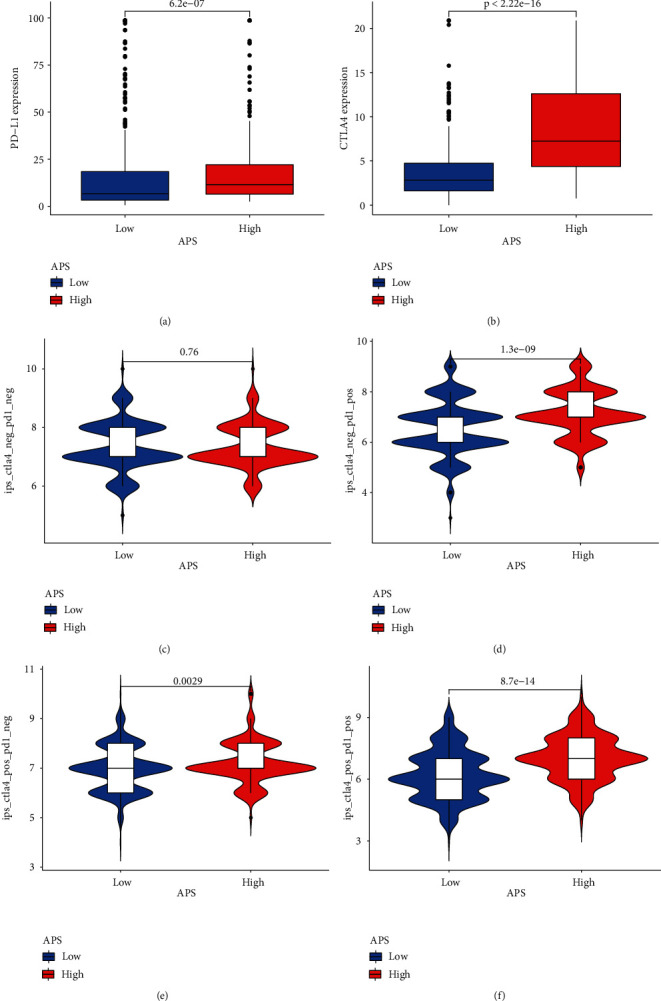
The effect of APS in predicting immunotherapy. (a, b) The expression of PD-L1 and CTLA4 between low and high APS groups. (c–f) The immunophenoscore (IPS) of the low and high APS groups with different immune checkpoint inhibitor (ICI) treatment. (c) CTLA4 (−)/PD1 (−); (d) CTLA4 (−)/PD1 (+); (e) CTLA4 (+)/PD1 (−); (f) CTLA4 (+)/PD1 (+).

Immunotherapy represented by anti-PD-1 and anti-CTLA4 has become one of the promising options for cancer treatment [27, 28]. Taking into account the differential enrichment of APS in the immune checkpoint pathway, we assessed whether APS can predict the response of LUSC patients to ICI treatment. Patients with high APS showed upregulation of PD-L1 and CTLA4, which indicated that APS can be used to predict the differential expression of related ICI (Figures 7(a) and 7(b)). Also consistent with expectations, whether anti-PD-1 or anti-CTLA4 alone or combination, patients in the high APS group had higher IPS (Figures 7(c)–7(f)), which means better clinical treatment response. The results suggested that APS can be used to predict the clinical response of LUSC patients to immunotherapy.

## 4. Discussion

Although in recent years, with the help of the imaging development and physical examination, more lung cancers have been treated at an early stage, there are still a considerable number of lung cancers, especially LUSC, which are already in the advanced stage when they are discovered [29]. At the same time, with recurrence and distant metastasis, surgical treatment still has great limitations for LUSC. As LUSC is a strongly heterogeneous cancer, radiotherapy, chemotherapy, and molecular targeted therapy cannot benefit all LUSC patients [6, 30]. Therefore, it is urgent to seek new molecular biomarkers to predict and guide the personalized treatment of LUSC. Previous studies have tried to explore different molecular subtypes in LUSC. For example, Wilkerson et al. divided 382 LUSC patients into four molecular subtypes based on mRNA levels [31]; Li et al. divided LUSC patients into seven subtypes based on 965 DNA methylation sites [32]; Xu et al. defined four molecular subtypes of LUSC based on DNA copy number or methylation-related gene expression [33]. Although these molecular subtypes tried to classify LUSC to find their prognostic markers, these classifications did not explore the differences in TME among subtypes and the targeted differences in prognostic treatment were not clear. Therefore, it is of great significance to carry out more accurate and comprehensive classification for LUSC clinical personalized treatment.

Autophagy plays different roles in different TME, and tumors can often change the growth and metastasis of tumors by regulating autophagy and thereby regulating the immune response [34, 35]. Therefore, further analysis of the differences in autophagy and immune pathways in different TME may help understand the specific mechanisms of tumor autophagy. The role of autophagy and autophagy-related genes has been explained in a variety of cancers. For example, a prognostic model based on autophagy-related genes was established in gastric cancer [36]; a prognostic model based on autophagy-related lncRNA was established in pancreatic cancer [37]; three colon cancer molecular subtypes were defined through the expression of autophagy-related genes [38]; similar research works have also been carried out to explore the relationship between autophagy and breast cancer, bladder cancer, and endometrial cancer [39–41]. However, there is a lack of research on autophagy in LUSC. Therefore, this study investigated the comprehensive role of autophagy in different LUSC phenotype and TME.

In this study, we comprehensively constructed the autophagy-related characteristics of LUSC through TCGA database and then divided LUSC patients into three different autophagy clusters through sixteen prognostic-related ARGs. Because tumors can regulate metabolism in TME through autophagy, regulate oxidative stress and hypoxia, and evade host immune surveillance to support cancer growth [42], we studied the relationship between three autophagy clusters and TME cell infiltration. Through ssGSEA, we can see that the TME cell infiltration in these three clusters showed different characteristics. Cluster A was rich in a large number of innate and adaptive immune cells. However, to our surprise, patients in cluster A did not show the expected survival advantage compared with the other two clusters. On the contrary, patients in cluster A had the worst prognosis. Previous studies have shown that there is a type of inflammatory tumor rich in infiltrating lymphocytes. In order to evade immune surveillance, such tumor recruits a large number of myeloid-derived immune cells or secrete factors including TGF-*β* to create an immunosuppressive tumor microenvironment. The tumor keeps immune cells around the tumor cell nest by producing highly expressed reactive mesenchyme and dense extracellular matrix, which makes the tumor exhibit strong immunosuppression despite the abundant immune cell infiltrating gallbladder [43–45]. Therefore, we focused on the TME stromal activation in cluster A, and the R package of ESTIMATE confirmed the significant increase in the stromal activity of the cluster. In the patients of cluster A, the expression of angiogenesis and EMT stromal signaling pathways and immune checkpoint molecules also increased significantly, which may limit the immune attack ability of the T cells. Meanwhile, it can be seen from the content of immune cells that cluster A contains more M2 macrophages, which was consistent with previous studies [46, 47]. For cluster C, although TME immune cells were poor, the patient's survival time was significantly longer than those of the other two clusters. From the GSVA enrichment analysis, patients in cluster C mainly activated the biological processes related to repair, including matrix repair and mismatch repair. The mesenchymal activation pathway also indicated that cluster C was also enriched in DNA damage repair. In addition, the expression of immune checkpoint molecule in this cluster was relatively low. Immune cell content analysis also showed that cluster C had a high proportion of quiescent mast cells and CD4 helper T cells. The better prognosis was also consistent with previous studies [46]. Therefore, we emphasized the role of repairing activated and functioning T cells in cluster C. Cluster B was characterized by relative immune suppression and relative stromal activation. These findings may promote our understanding of the relationship between tumor autophagy, TME cell infiltration, and matrix repair. Next, we reclassified LUSC by DEGs between the three clusters to obtain three gene clusters. The three gene clusters also had obvious heterogeneity, and their prognosis had obvious differences. From the mulberry diagram, it can be seen that the autophagy clusters had a rough correspondence with the autophagy gene clusters. Taking into account the heterogeneity of LUSC, we constructed APS model based on these prognostic-related DEGs to further evaluate the autophagy characteristics of each LUSC patient. Cluster C and gene cluster C had low APS, corresponding to a better prognosis, and their autophagy gene expression was at a low level. Cluster A and most gene cluster A had high APS, corresponding to a poor prognosis, and their autophagy gene expression was at a high level. The independent prognostic value of APS in LUSC patients has been further confirmed. Then we observed a negative correlation between APS and TMB, which also means that lower autophagy gene expression has higher TMB, corresponding to a better prognosis. At the same time, the expression of immune checkpoint molecules like PD-L1 and CTLA4 was positively correlated with APS, indicating that APS may have a guiding role in predicting immunotherapy. In the TCIA database, significant clinical benefits were observed in patients with high APS who received ICI therapy, which may provide new guidance for future immunotherapy of LUSC patients. In conclusion, this study revealed that the three autophagy clusters have different autophagy and TME infiltration characteristics in LUSC, and the APS model constructed at the same time can be used as a biomarker to predict patient prognosis and guide immunotherapy.

## Data Availability

The datasets generated and/or analyzed during this study are available from GEO (https://www.ncbi.nlm.nih.gov/geo/) and TCGA official website (https://portal.gdc.cancer.gov/repository).

## Supplementary Materials

Supplementary Materials Figure S1: identification of autophagy clusters in LUSC. (A) Consensus clustering cumulative distribution function (CDF) with the number of subtypes *k* = 2 to 9. ((B)–(D)) Unsupervised clustering of prognostic-related ARGs in LUSC and consensus matrices for *k* = 2,4,5. (E) The relative expression of 16 prognostic-related ARGs in three autophagy clusters. Figure S2: immune cell characteristics in the three autophagy clusters. (A) The relative percentages of 22 different immune subsets in each LUSC patient. (B) Difference in immune score among three clusters in LUSC. (C) Difference in stromal score among three clusters in LUSC. (D) Difference in estimate score among three clusters in LUSC. (E) The relationship between 22 immune cells. Figure S3: identification of autophagy gene clusters in LUSC. (A) 373 differentially expressed genes (DEGs) between three autophagy clusters were shown in the Venn diagram. (B) Consensus clustering CDF with the number of subtypes *k* = 2 to 9. (C) Delta area curve for clustering, representing the relative change in area under the CDF curve. ((D)–(F)) Unsupervised clustering of prognostic-related DEGs in LUSC and consensus matrices for *k* = 2, 4, 5. (G) PCA for the three gene clusters among LUSC patients. Figure S4: evaluation of the prognosis and immune value of the APS model. (A) Survival analysis of APS in GSE73403 cohort. (B) Survival analysis of APS in GSE157011 cohort. (C) Survival analysis of APS in GSE74777 cohort. (D) Differences in stroma-activated pathways between low and high APS groups. The asterisks represent the *P* value (^*∗*^*P* < 0.05; ^*∗∗*^*P* < 0.01; ^*∗∗∗*^*P* < 0.001). (E) The difference in the percentage of survival status (Fustat) between the low and high APS groups. (F) The APS in different survival status (Fustat) of LUSC patients. Figure S5: the relationship between APS and clinicopathological characteristics. The clinicopathological features of LUSC include (A) TNM stage, (B) T stage, (C) N stage, and (D) M stage. Figure S6: covariance of principal components.
