# Supplementary material for: Characteristic of Molecular Subtypes in Lung Squamous Cell Carcinoma Based on Autophagy-Related Genes and Tumor Microenvironment Infiltration
Source: J Oncol. 2022 Sep 13;2022:3528142. doi: 10.1155/2022/3528142 (PMC9489399; doi:10.1155/2022/3528142)
Supplement: Supplementary Materials — Figure S1: identification of autophagy clusters in LUSC. (A) Consensus clustering cumulative distribution function (CDF) with the number of subtypes k = 2 to 9. ((B)–(D)) Unsupervised clustering of prognostic-related ARGs in LUSC and consensus matrices for k = 2,4,5. (E) The relative expression of 16 prognostic-related ARGs in three autophagy clusters. Figure S2: immune cell characteristics in the three autophagy clusters. (A) The relative percentages of 22 different immune subsets in each LUSC patient. (B) Difference in immune score among three clusters in LUSC. (C) Difference in stromal score among three clusters in LUSC. (D) Difference in estimate score among three clusters in LUSC. (E) The relationship between 22 immune cells. Figure S3: identification of autophagy gene clusters in LUSC. (A) 373 differentially expressed genes (DEGs) between three autophagy clusters were shown in the Venn diagram. (B) Consensus clustering CDF with the number of subtypes k = 2 to 9. (C) Delta area curve for clustering, representing the relative change in area under the CDF curve. ((D)–(F)) Unsupervised clustering of prognostic-related DEGs in LUSC and consensus matrices for k = 2, 4, 5. (G) PCA for the three gene clusters among LUSC patients. Figure S4: evaluation of the prognosis and immune value of the APS model. (A) Survival analysis of APS in GSE73403 cohort. (B) Survival analysis of APS in GSE157011 cohort. (C) Survival analysis of APS in GSE74777 cohort. (D) Differences in stroma-activated pathways between low and high APS groups. The asterisks represent the P value (∗P < 0.05; ∗∗P < 0.01; ∗∗∗P < 0.001). (E) The difference in the percentage of survival status (Fustat) between the low and high APS groups. (F) The APS in different survival status (Fustat) of LUSC patients. Figure S5: the relationship between APS and clinicopathological characteristics. The clinicopathological features of LUSC include (A) TNM stage, (B) T stage, (C) N stage, and (D) M stage. Figure S6: covarian [file 3528142.f1.docx]

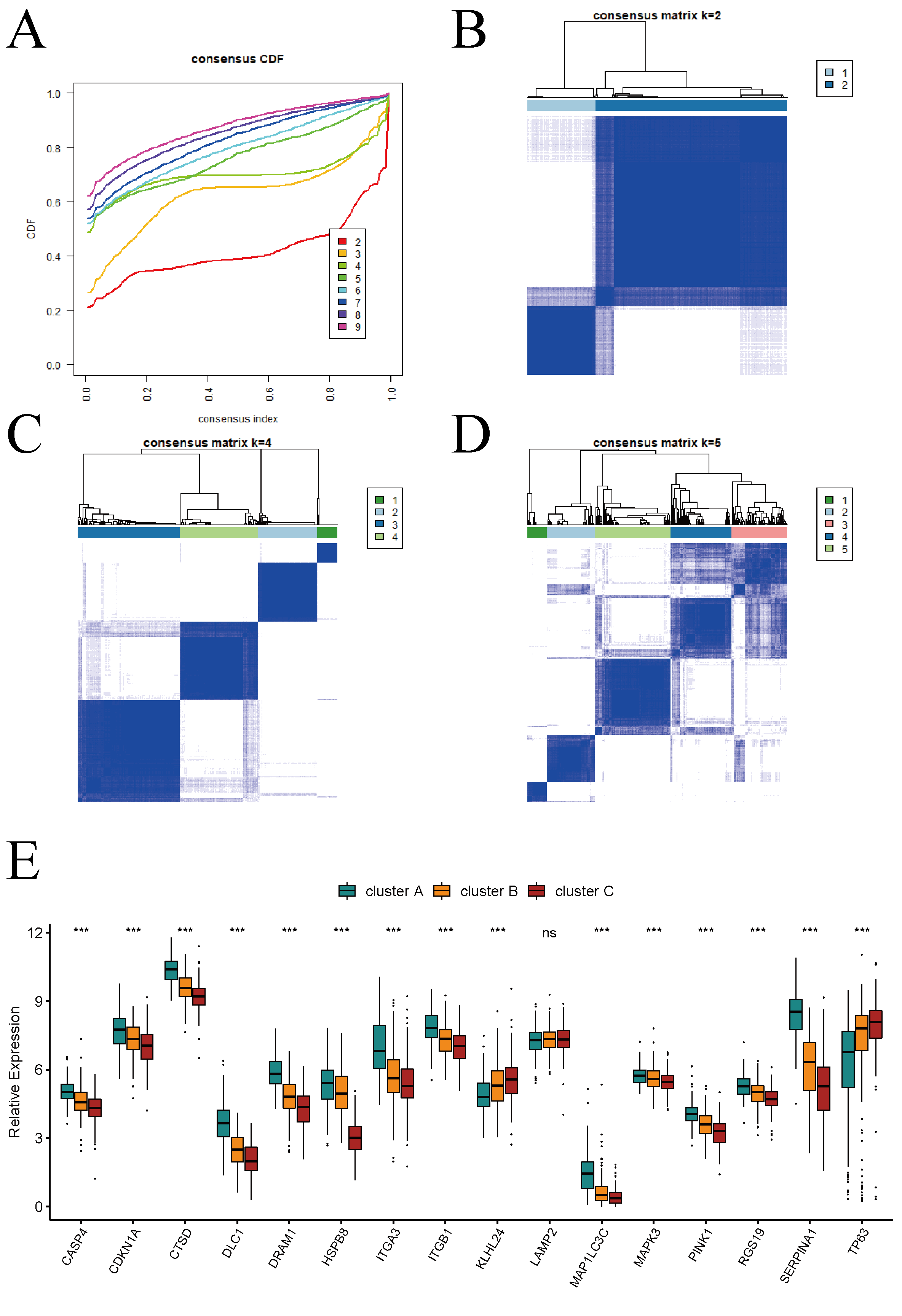


Figure S1. Identification of autophagy clusters in LUSC. (A) Consensus clustering cumulative distribution function (CDF) with the number of subtypes k = 2 to 9. (B-D) Unsupervised clustering of prognostic-related ARGs in LUSC and consensus matrices for k = 2,4,5. (E) The relative expression of 16 prognostic-related ARGs in three autophagy clusters.


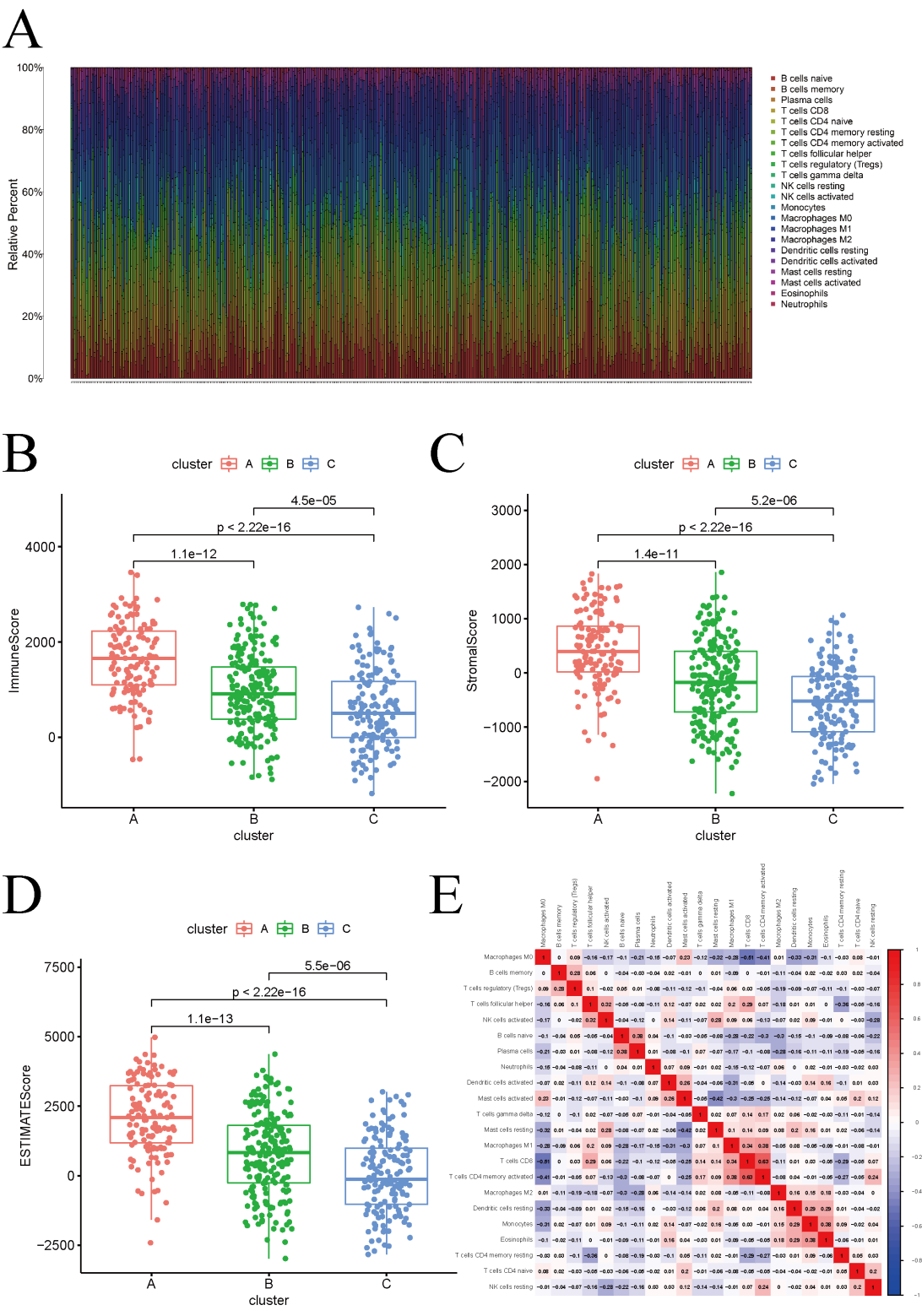


Figure S2. Immune cell characteristics in the three autophagy clusters. (A) The relative percentages of 22 different immune subsets in each LUSC patient. (B) Difference in immune score among three clusters in LUSC. (C) Difference in stromal Score among three clusters in LUSC. (D) Difference in estimate score among three clusters in LUSC. (E) The relationship between 22 immune cells.


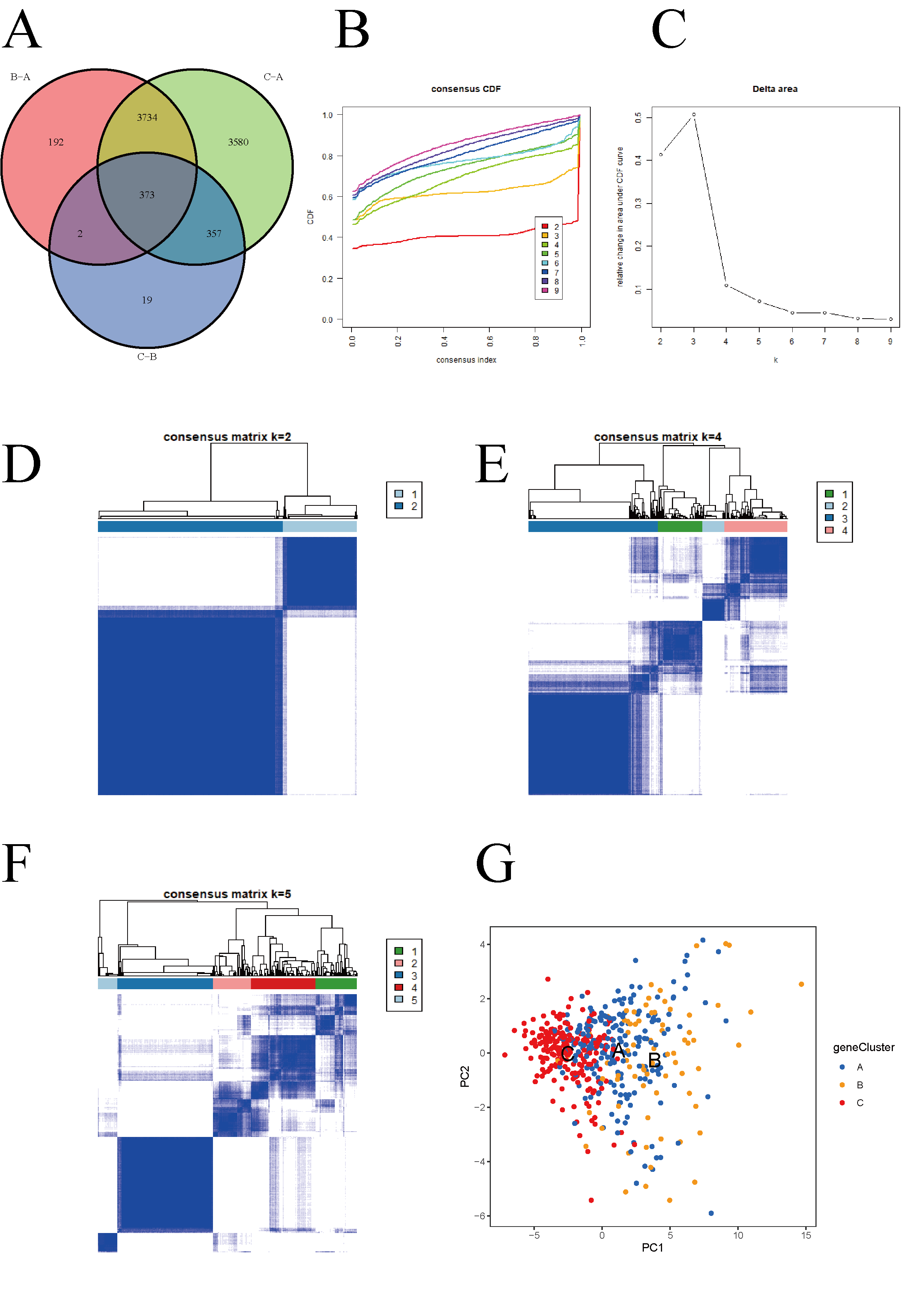


Figure S3. Identification of autophagy gene clusters in LUSC. (A) 373 differentially expressed genes (DEGs) between three autophagy clusters were shown in the Venn diagram. (B) Consensus clustering CDF with the number of subtypes k = 2 to 9. (C) Delta area curve for clustering, representing the relative change in area under the CDF curve. (D-F) Unsupervised clustering of prognostic-related DEGs in LUSC and consensus matrices for k = 2,4,5. (G) PCA for the three gene clusters among LUSC patients.


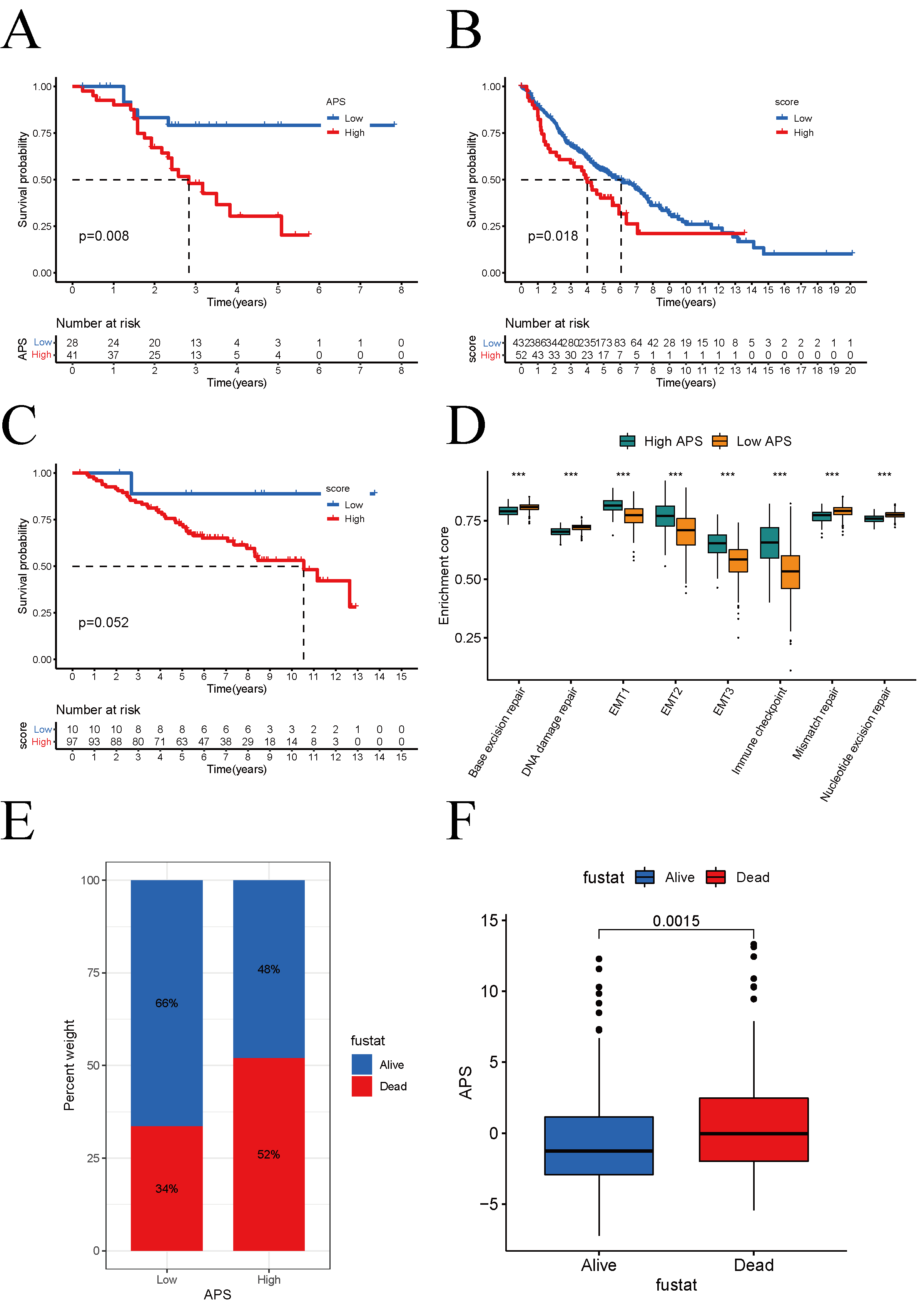


Figure S4. Evaluation of the prognosis and immune value of the APS model. (A) Survival analysis of APS in GSE73403 cohort. (B) Survival analysis of APS in GSE157011 cohort. (C) Survival analysis of APS in GSE74777 cohort. (D) Differences in stroma-activated pathways between low and high APS groups. The asterisk represented the P value (*P < .05; **P < .01; ***P < .001). (E) The difference in the percentage of survival status (Fustat) between the low and high APS groups. (F) The APS in different survival status (Fustat) of LUSC patients.


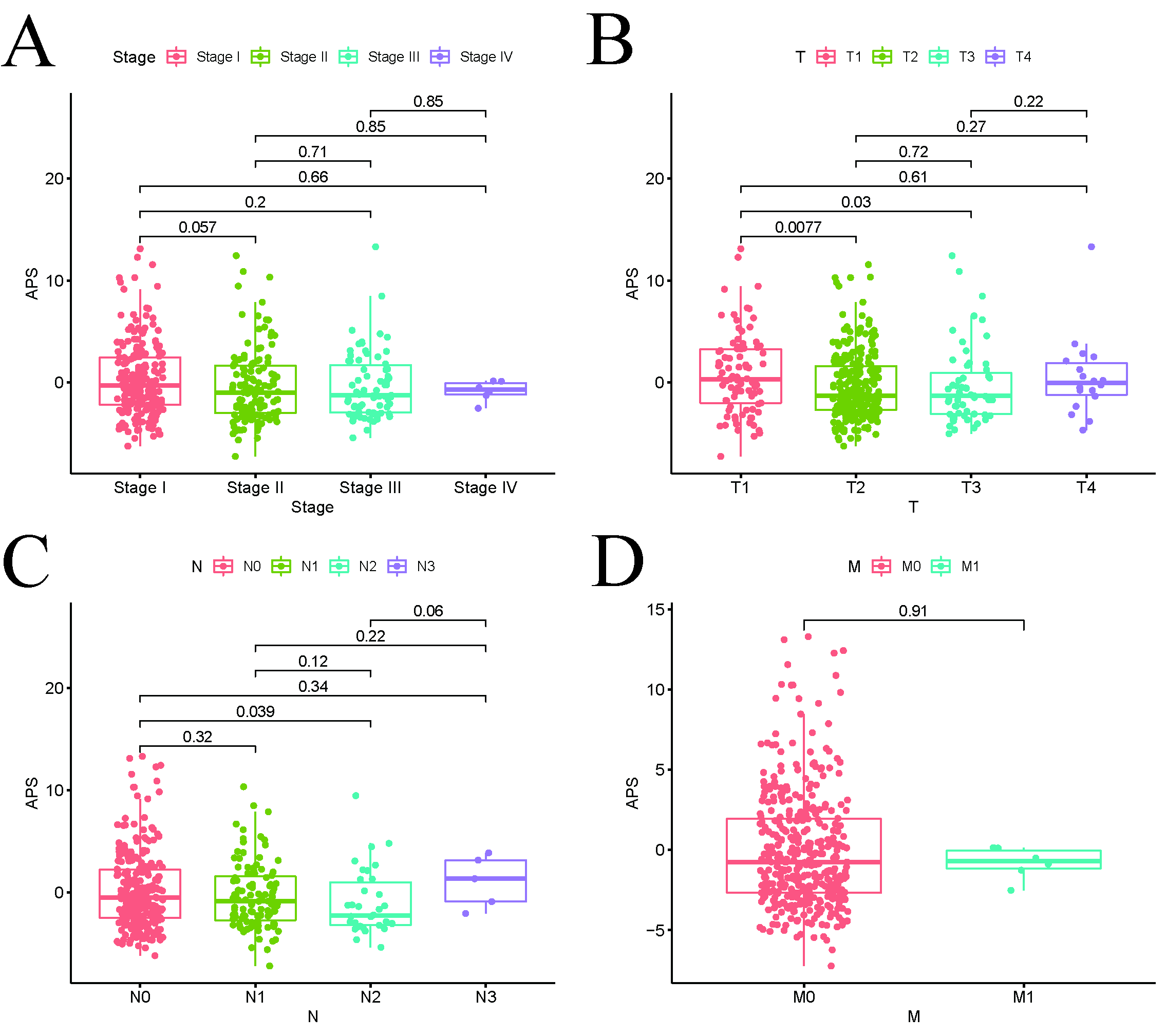


Figure S5. The relationship between APS and clinicopathological characteristics. The clinicopathological features of LUSC include: (A) TNM stage, (B) T stage, (C) N stage, (D) M stage.


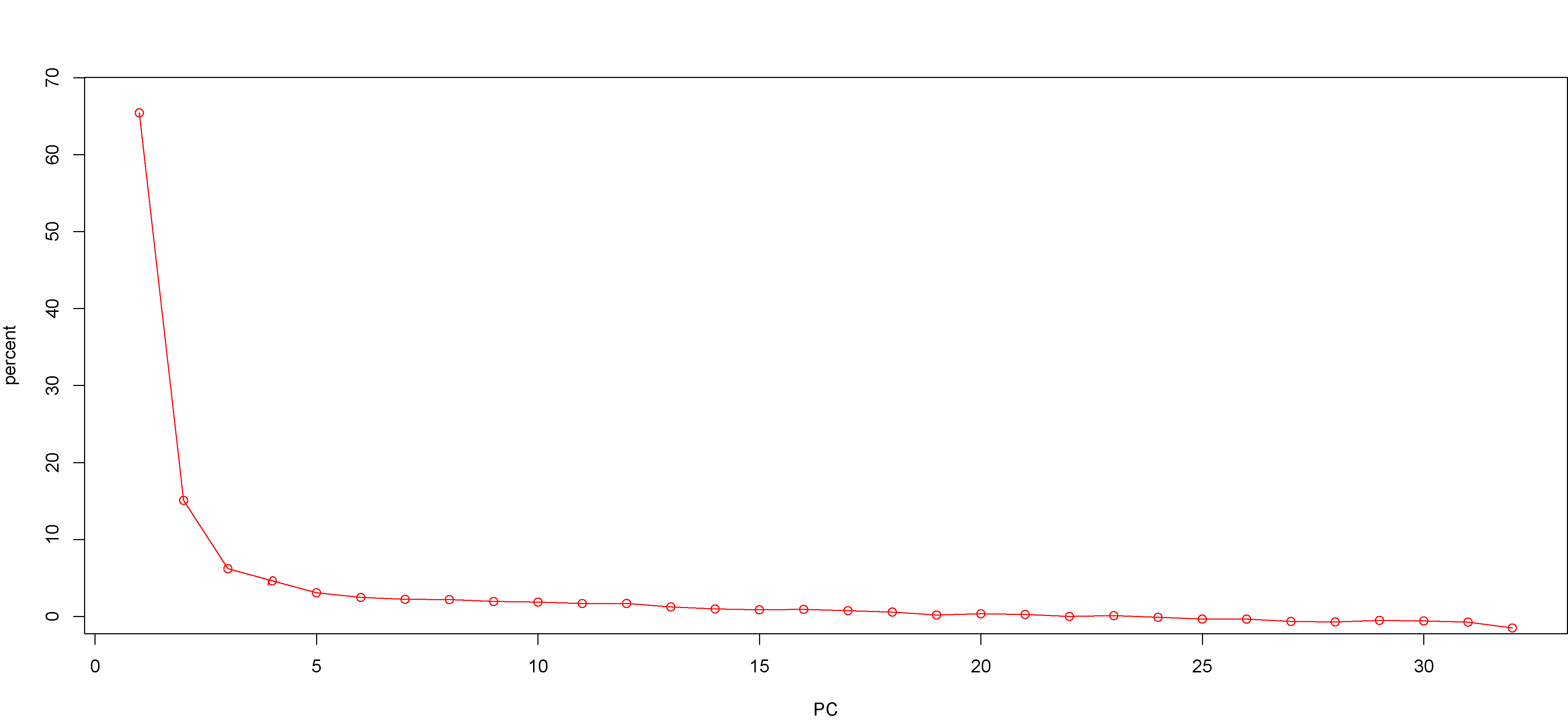


Figure S6. Covariance of principal components.
